# Processing fluency hinders subsequent recollection: an electrophysiological study

**DOI:** 10.3389/fpsyg.2015.00863

**Published:** 2015-06-24

**Authors:** Bingbing Li, Chuanji Gao, Wei Wang, Chunyan Guo

**Affiliations:** Beijing Key Laboratory of Learning and Cognition, Department of Psychology, College of Education, Capital Normal UniversityBeijing, China

**Keywords:** processing fluency, masked repetition priming, recollection, familiarity, ERP

## Abstract

Although many behavioral studies have investigated the effect of processing fluency on subsequent recognition memory, little research has examined the neural mechanism of this phenomenon. The present study aimed to explore the electrophysiological correlates of the effects of processing fluency on subsequent recognition memory by using an event-related potential (ERP) approach. The masked repetition priming paradigm was used to manipulate processing fluency in the study phase, and the R/K paradigm was utilized to investigate which recognition memory process (familiarity or recollection) was affected by processing fluency in the test phase. Converging behavioral and ERP results indicated that increased processing fluency impaired subsequent recollection. Results from the analysis of ERP priming effects in the study phase indicated that increased perceptual processing fluency of object features, reflected by the N/P 190 priming effect, can hinder encoding activities, reflected by the LPC priming effect, which leads to worse subsequent recollection based recognition memory. These results support the idea that processing fluency can influence subsequent recognition memory and provide a potential neural mechanism underlying this effect. However, further studies are needed to examine whether processing fluency can affect subsequent familiarity.

## Introduction

Processing fluency refers to the ease or difficulty of current cognitive processing (for a review, see Oppenheimer, 2008). Although processing fluency should improve subsequent recognition memory according to intuition and some cognitive theories (e.g., dual-store model of memory, Atkinson and Shiffrin, 1968; for a similar argument, see Hirshman and Mulligan, 1991), experimental studies have produced the opposite results. For example, studies of the perceptual interference effect have shown that more-easily perceived items (i.e., presented with no backward mask) were associated with worse subsequent recognition memory (in recall and recognition tests) compared to items presented with backward masking to interfere with perception (Nairne, 1988; Hirshman and Mulligan, 1991; Hirshman et al., 1994; Mulligan, 1996, 1998; Besken and Mulligan, 2013). Studies using other methods to manipulate processing fluency (e.g., presentation of text in fonts that are easy or difficult to read) have also shown that more-fluently processed items were associated with worse subsequent recognition memory (e.g., Diemand-Yauman et al., 2011; Sungkhasettee et al., 2011). Although numerous behavioral studies have provided evidence supporting the effect of processing fluency on subsequent recognition memory, the underlying neural mechanism of this effect remains unknown.

The present study aimed to investigate the electrophysiological correlates of the effects of processing fluency on subsequent recognition memory by recording event-related potential (ERP) responses. Unlike previous studies, most of which altered the superficial features (e.g., font, Diemand-Yauman et al., 2011) of presented items to manipulate processing fluency, the present study used the masked repetition priming paradigm to manipulate fluency in the study phase. In a typical masked repetition priming experiment, a masked prime item, which is either the same as or unrelated to the target item, is presented briefly before the target item. Such briefly presented prime items are thought to facilitate the processing of target items, despite the subjects’ unawareness of its presentation (e.g., Jacoby and Whitehouse, 1989; Woollams et al., 2008). The advantage of the masked repetition priming paradigm is that subjects are unaware of the source of fluency during the experiment, which can mitigate the influence of subjective strategy with respect to items of different fluency magnitudes.

The masked priming paradigm has been used widely in studies investigating the contribution of fluency in the test phase to recognition memory (e.g., Jacoby and Whitehouse, 1989; Rajaram, 1993; Westerman, 2001, 2008; Westerman et al., 2002, 2003; Kurilla and Westerman, 2008). However, few studies have utilized this paradigm in the study phase to investigate the effect of fluency on subsequent recognition memory. Another difference between the present and previous studies is that we used pictures as stimuli. This approach aided the examination of whether the effect of processing fluency on subsequent recognition memory can be extended to picture stimuli.

Event-related potential studies investigating masked repetition priming effects using pictures have provided some insight into the electrophysiological correlates of picture processing fluency (Eddy et al., 2006; Eddy and Holcomb, 2010). These priming effects have been associated with three ERP components, N/P190, N300, and N400, which are associated with processing at multiple levels. The N/P190 priming effect, which is negative in anterior regions and positive in occipital regions, occurs at 100–250 ms post–stimulus onset. This effect is less negative in anterior regions and more positive in posterior regions for primed pictures, and is presumed to reflect earlier perceptual processing of object features. N300, which occurs around 250–300 ms, is a typical component for pictures. It is less negative for primed than for unprimed pictures, and is thought to reflect object-specific representation processing. N400, which is distributed widely, occurs around 300–500 ms. It is also less negative for primed than for unprimed pictures, and is thought to reflect general semantic processing (Eddy et al., 2006).

Later priming effects [e.g., late positive component (LPC)] have rarely been reported in masked repetition priming studies using pictures. However, the results of ERP studies have suggested that episode encoding activities are associated with relatively later ERPs (e.g., LPC). The ERPs of subsequently remembered items are more positive than those of subsequently forgotten items at 400–800 ms post–stimulus onset. This phenomenon is referred to as the difference due to subsequent memory (DM) effect (e.g., Paller et al., 1987; Paller and Wagner, 2002; Nie et al., 2004). Although some studies have reported differences in ERPs between subsequently remembered and forgotten items in earlier time windows (e.g., 300–500 ms), these effects are thought to reflect other (e.g., semantic) processing during the encoding phase and may not be predictive of subsequent memory (e.g., Cansino et al., 2010; Kuo et al., 2012). As processing at different levels is associated with temporally distinct ERP components, we can determine the level at which processing (e.g., early perceptual or later encoding) affects subsequent recognition memory by investigating the ERP priming effects in the study phase.

Recognition memory is not a unitary process. It is supported by two distinct processes – familiarity and recollection – as suggested by dual-process models of recognition memory. Recollection refers to the recognition of a prior event with recall of context or other relevant information, whereas familiarity refers to the recognition of a prior event with no such recall (Mandler, 1980; Yonelinas, 2002; Aggleton and Brown, 2006). Few studies have directly investigated whether processing fluency in the encoding phase affects subsequent familiarity and recollection to the same extent, which could improve the understanding not only of how processing fluency affects subsequent recognition memory, but also of the relationship between familiarity and recollection. We utilized the remember/know (R/K) paradigm (Tulving, 1985), which has been used widely to investigate recollection and familiarity (Migo et al., 2012), in the test phase to investigate the effects of processing fluency on subsequent recollection and familiarity specifically.

Monitoring ERPs can also aid in the determination of which recognition memory process is affected by processing fluency. Previous studies have found that spatiotemporally distinct ERP old/new effects are associated with familiarity and recollection. The FN400 old/new effect, which is distributed in the frontal-central region at around 300–500 ms post–stimulus onset, has been thought to be correlated with familiarity, although some researchers have recently claimed that it is correlated instead with conceptual priming (Paller et al., 2007). The LPC old/new effect, which is distributed in the central-parietal region at around 500–800 ms post–stimulus onset, is correlated with recollection (Rugg et al., 1998; Curran, 2000; for a review, see Rugg and Curran, 2007). Thus, we can investigate which recognition memory process is affected by processing fluency by comparing the FN400 and LPC effects of primed and unprimed studied items in the test phase.

In the present study, we employed the masked repetition priming paradigm in the study phase and the R/K paradigm in the test phase to investigate which subsequent recognition memory process is affected by processing fluency in the study phase. ERPs in the encoding and test phases were recorded to explore the neural mechanism(s) underlying the effects of processing fluency on subsequent recognition memory.

## Materials and Methods

### Participants

Eighteen students (13 female, 19–25 years old, all right handed) from Capital Normal University, participated in this experiment. All participants had normal (or corrected-to-normal) vision and no neurological illness. Data from two participants were not included in the analysis due to excessive muscle artifacts and electrode drift in more than 25% of trials. All participants signed an informed consent form and were paid for their participation. The Human Research Ethics Committee of Capital Normal University approved this research.

### Materials

Stimuli were 400 color pictures (screen size, 200 × 200 pixels) from the Hemera Photo-Objects database (*n* = 340) and the internet (*n* = 60). Pictures were edited with Photoshop software to be similar in size and to have white backgrounds with no extra features. The old/new and priming/unpriming statuses of the picture sets were counterbalanced across participants. An additional 24 pictures were used in filler and practice trials, as described below.

### Procedure

The experiment consisted of an incidental study phase and a test phase (consisting of two test blocks). In the study phase, participants were asked to judge whether depicted items were living or non-living. A 16-picture practice block was administered before the formal study block. The study block involved the presentation of 200 pictures, with two filler pictures presented at the beginning and end of the block to avoid primacy and recency effects. The presentation of each picture was preceded by the brief presentation of a masked prime picture, which was either the same as (primed trials, 50%) or unrelated to (another unrelated picture in unprimed condition, unprimed trials, 50%) the target picture. The masked repetition priming procedure was based on those used in previous studies (Eddy et al., 2006; Lucas et al., 2012) and the focus of our study.

Participants were asked to write down as many place names as possible for 3 min immediately after the study phase, to avoid the ceiling effect of performance level in the test blocks. They were then told about the surprise memory test and given instructions for R/K/New responses. A practice test with 10 pictures (six from the practice block, four new) was administered before the test phase. During the practice test, participants were asked to report why they made R or K responses to ensure that they understood the instructions and did not confuse R and K responses with confidence ratings. Each test block consisted of the presentation of 200 pictures (50% studied, 50% unstudied). Participants were asked to report their subjective memory experiences about each picture by responding ‘R’ if they could recall any information associated with the test picture (e.g., their feeling when they saw the picture or what the picture looked like on the screen), ‘K’ if they could not recall any such information but felt that they had seen the picture in the study phase, or ‘New’ if they felt that they had not seen the picture in the study phase.

Stimuli were presented against a white background in the center of a 17″ CRT monitor (1024 × 768 resolution, 85-Hz refresh rate) positioned ∼70 cm in front of the participant. In the study phase, each trial began with a cross fixation presented randomly between 1506 and 2000 ms. A forward mask was then presented for 306 ms, followed by a prime picture for 35 ms and a backward mask for 70 ms. Immediately thereafter, the target picture was presented for 1506 ms. The forward and backward mask was a kaleidoscopic picture selected from the stimuli of Voss and Paller (2010). Participants were not informed about the presentation of the masked pictures during the experiment. They were told that the flickering kaleidoscopic pictures were presented to obtain baseline electroencephalographic (EEG) data. In the test phase, each trial began with a cross fixation presented randomly between 1506 and 2000 ms, followed by presentation of the test picture for 506 ms, and then a blank screen for 2000 ms, during which participants made the R/K/New judgment.

Electroencephalographics were recorded with 64 Ag/AgCl electrodes positioned in a nylon electrode cap by the Neuro Scan system. EEGs were recorded with a band pass of 0.05–100 Hz (0.05–30 Hz filtered oﬄine), and sampled at a rate of 500 Hz. All channels were referred to the left mastoid electrode and re-referenced to averaged mastoids in oﬄine analysis. Electrodes were placed above and below the center of left eye and on the canthi to record vertical and horizontal electro-oculograms. EOG blink artifacts were corrected using a linear regression estimate (Semlitsch et al., 1986). Electrode impedance was kept below 5 kΩ. EEGs were segmented into epochs from 100 ms prior to stimulus onset (for baseline correction) to 900 ms after stimulus onset. Epochs containing artifacts exceeding ± 75 μV were excluded from ERPs analyzing.

Two midline electrode clusters were selected in the analysis of the ERPs. The clusters were frontal: F3, FZ, and F4 and parietal: P3, PZ, and P4. Statistical comparisons were performed using repeated-measures ANOVA (criterion *p* = 0.05). Greenhouse-Geisser correction was used where appropriate (uncorrected degrees of freedom were reported with corrected *p*-values in the results section). Bonferroni-correction was used in *post hoc* comparisons.

## Results

### Behavioral Data

#### Study Phase

To examine the effect of masked repetition priming on performance, we used paired *t*-tests to analyze the reaction times (RTs) and accuracies for living/non-living judgments of primed/unprimed pictures. RTs to primed pictures were faster than unprimed pictures [*M* = 680 ms, SE = 16 vs. *M* = 702 ms, SE = 15, *t*(15) = 5.603, *p* < 0.001, SE = 4.06], which indicated that masked priming facilitated the processing (i.e., increased the processing fluency) of primed pictures. However, the difference between the accuracies of primed and unprimed pictures was not significant [*M* = 0.98, SE = 0.003 vs. *M* = 0.97, SE = 0.005, *t*(15) = 1.678, *p* = 0.114, SE = 0.006).

#### Test Phase

**Table 1** depicts the raw proportions of responses in each condition. Most dual-process models claim that familiarity and recollection are either independent or redundant (for review, see Yonelinas, 2002). In the R/K procedure, subjects are asked to respond K when an item is ‘familiar but not recollected’ rather than when the item is ‘familiar.’ Consequently, the proportion of K responses underestimates the actual probability of familiarity in R/K procedure (Wagner et al., 1997; Yonelinas, 2002). To compensate for this underestimation, Yonelinas and Jacoby (1995) proposed the independent remember/know procedure (IK procedure), in which familiarity (i.e., IK) is calculated as proportion of K responses/(1 – proportion of R responses). We used the IK procedure to obtain an unbiased estimate of familiarity in our analysis of the effects of priming on subsequent recollection and familiarity.^1^

**Table 1:** Mean proportions (in percentage, with SE in parentheses) of each response type to studied (primed and unprimed in the study phase) and unstudied pictures.

Study status	Prime status	Remember	Know	New
Studied	Primed	38(4)	36(4)	26(3)
	Unprimed	41(4)	33(4)	26(3)
Unstudied		3(1)	13(2)	84(3)

Overall accuracy (Pr, calculated as the proportion of Hits minus the proportion of False Alarms; Snodgrass and Corwin, 1988) was 0.36 (SE = 0.036) for R and 0.43 (SE = 0.031) for IK. The Pr values of R and IK were both greater than zero [*t*(15) = 10.071, *p* < 0.001, SE = 0.036, and *t*(15) = 13.073, *p* < 0.001, SE = 0.031, respectively]. These results suggest that the memory performance was above the level of chance.

To investigate the effect of masked repetition priming on subsequent recollection and familiarity, we conducted a two-way ANOVA involving response type (R/IK) and prime status (primed/unprimed in the study phase) on the proportions of R and IK to studied items. The results revealed a significant two-way interaction [*F*(1,15) = 4.965, *p* = 0.042, MSE = 0.002]. Proportions of R responses to unprimed pictures were significantly greater than primed pictures [*t*(15) = 2.303, *p* = 0.036, SE = 0.016], whereas the proportions of IK were not significantly different between primed and unprimed pictures [*M* = 0.56, SE = 0.04 vs. *M* = 0.54, SE = 0.04; *t*(15) = 1.023, *p* = 0.322, SE = 0.018].^2^ These results indicate that masked repetition priming affected subsequent recollection but not familiarity.

### ERP Data

#### Study Phase

Based on previous studies (e.g., Eddy et al., 2006) and the observation of ERP waveforms, a time-window of 100–250 ms and a time window of 500–700 ms were selected to index the masked priming effects.^3^ The ERP polarity and the priming effect at 150–250 ms were reversed in occipital regions, which was similar to previous studies (e.g., Eddy et al., 2006). However, as we focused on ERPs at frontal and parietal regions to investigate the priming and memory effects and the amplitudes and priming effect at occipital regions during this time-window were relatively small compared to anterior regions as the topographic map showed, we termed ERPs during this time-window as N/P 190 to follow the literature and did not include electrodes at occipital regions in the statistical analysis.

##### Overall priming effects

**FIGURE 1:**
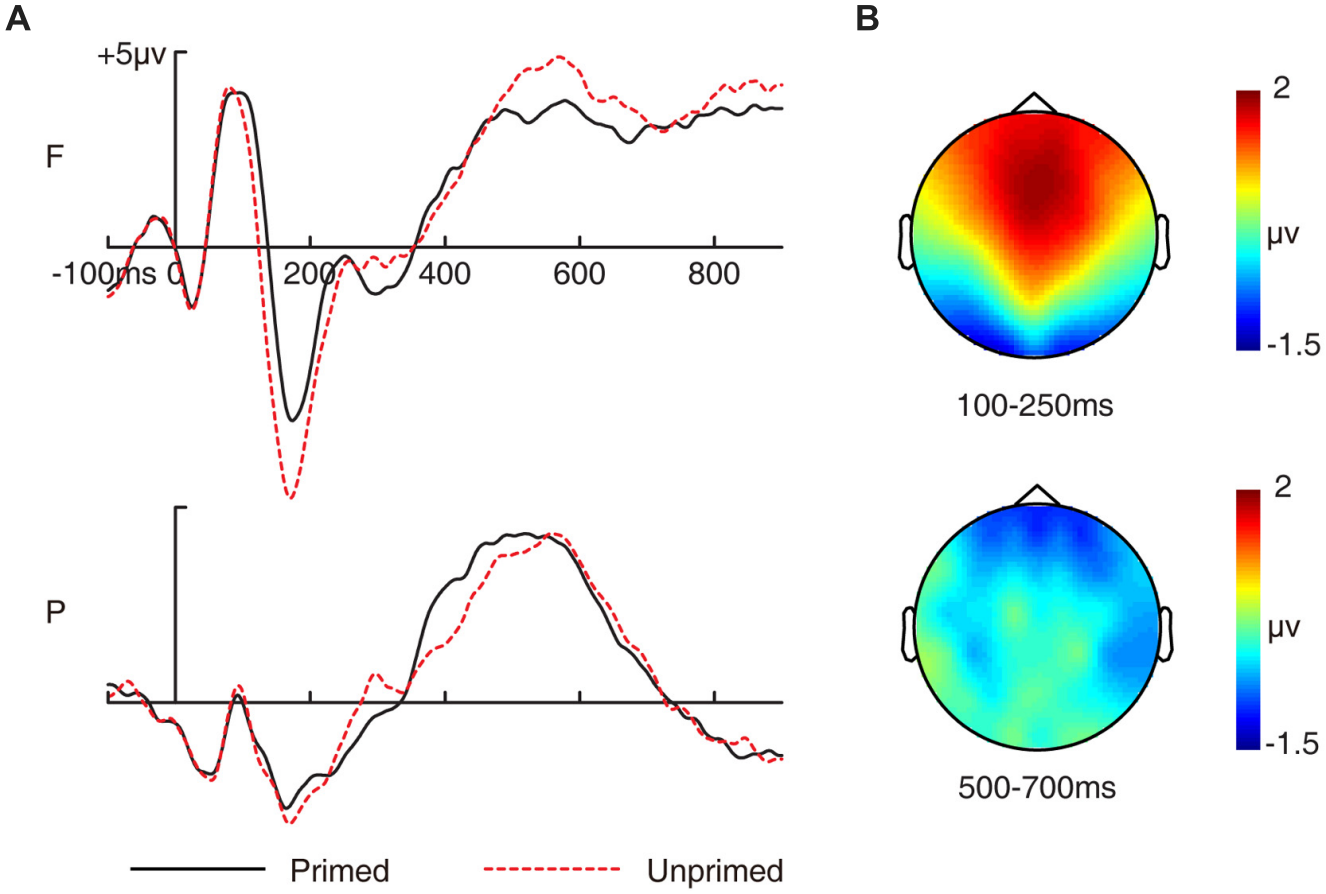
**Grand-averaged event-related potential (ERP) waveforms and topographic maps for priming effects in the study phase. (A)** Grand-averaged ERP waveforms for primed and unprimed pictures in the study phase. **(B)** Topographic maps for the overall priming effects (ERPs to primed pictures minus ERPs to unprimed pictures) for 300–500 ms and 500–700 ms. F, frontal electrode cluster; P, parietal electrode cluster.

Grand-averaged ERP waveforms for primed and unprimed pictures and the topographic maps of the overall priming effect (ERPs for primed pictures minus ERPs for unprimed pictures) for 100–250 ms and 500–700 ms are shown in **Figures 1A,B**. ERPs were averaged for primed and unprimed pictures without considering subsequent memory to investigate the overall priming ERP effects associated with masked repetition priming. Two-way ANOVA involving prime status (primed/unprimed) and electrode cluster (frontal/parietal) was conducted for primed and unprimed pictures for this analysis.

###### 100–250 ms

The two-way ANOVA revealed a significant two-way interaction [*F*(1,15) = 35.296, *p* < 0.001, MSE = 0.217]. Amplitudes of unprimed pictures were more negative than primed pictures at frontal [*F*(1,15) = 33.299, *p* < 0.001, MSE = 0.581] but not at parietal electrode cluster [*F*(1,15) = 0.534, *p* = 0.476, MSE = 0.44]. Thus, this priming effect was anterior distributed as depicted in **Figure 1B**.

###### 500–700 ms

The two-way ANOVA revealed a significant two-way interaction [*F*(1,15) = 12.1, *p* = 0.003, MSE = 0.207]. Amplitudes of unprimed pictures were more positive than primed pictures at frontal [*F*(1,15) = 4.717, *p* = 0.046, MSE = 1.39] but not at parietal electrode cluster [*F*(1,15) = 0.116, *p* = 0.738, MSE = 0.879]. Thus, this priming effect was also anterior distributed as depicted in **Figure 1B**.

##### Repetition priming effects as a function of subsequent memory

**FIGURE 2:**
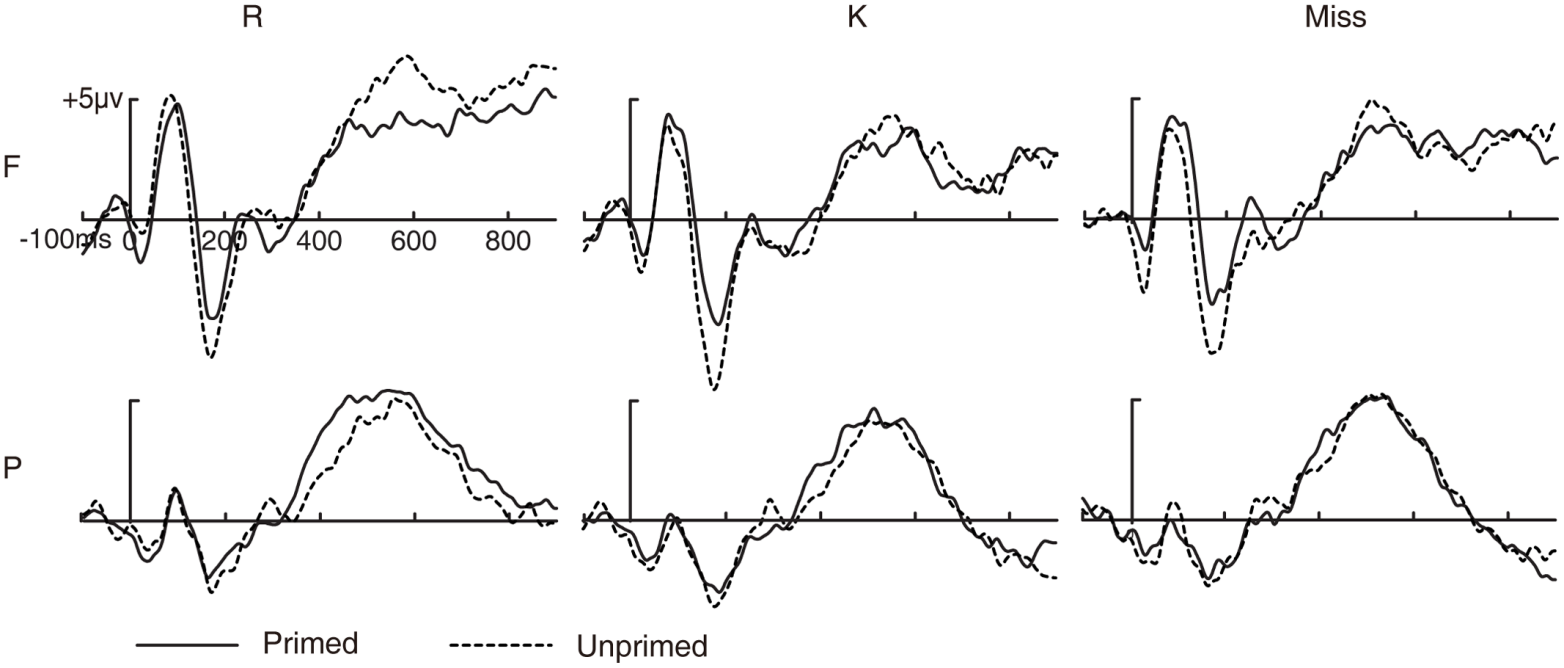
**Event-related potential waveforms for primed and unprimed pictures as a function of subsequent memory.** Grand-averaged ERP waveforms for primed and unprimed pictures as a function of subsequent recognition memory. R, pictures with subsequent R responses; K, pictures with subsequent K responses; Miss, pictures with subsequent New responses; F, frontal electrode cluster; P, parietal electrode cluster.

To investigate which repetition priming effect is related to the priming effect on subsequent recognition memory, we averaged ERPs to primed/unprimed pictures as a function of subsequent memory (R/K/Miss). If the ERP priming effect is related to the priming effect on subsequent recognition memory, there should be an interaction between subsequent memory and prime status. Thus, three-way ANOVA, involving subsequent memory (R/K/Miss), prime status (primed/unprimed) and electrode cluster (frontal/parietal), was conducted on ERPs to primed and unprimed pictures as a function of subsequent memory. Data from two subjects were excluded in this analysis because they had fewer than 16 artifact-free trials under one or more conditions. **Figure 2** shows the ERP waveforms for primed/unprimed pictures as a function of subsequent memory.

###### 100–250 ms

The three-way ANOVA revealed a significant main effect of subsequent memory [*F*(2,26) = 5.137, *p* = 0.017, MSE = 1.729] and a significant two-way interaction between prime status and electrode cluster [*F*(1,13) = 22.702, *p* < 0.001, MSE = 0.953], but no other significant two- or three-way interactions involving subsequent memory (all *p* > 0.1). These results suggest that the priming effect during this time window was similar across pictures with subsequent R, K and New responses and, hence, was not predictive of priming effect on subsequent memory.

###### 500–700 ms

The three-way ANOVA revealed a significant main effect of subsequent memory [*F*(2,26) = 9.772, *p* = 0.001, MSE = 4.13], a significant two-way interaction between prime status and electrode cluster [*F*(1,13) = 12.87, *p* = 0.003, MSE = 0.995] and a significant three-way interaction [*F*(2,26) = 3.523, *p* = 0.049, MSE = 1.912]. The two-way interaction between prime status and electrode cluster was significant for subsequent R responses [*F*(1,13) = 10.841, *p* = 0.006, MSE = 2.121], but not for subsequent K and New responses [K: *F*(1,13) = 3.476, *p* = 0.085, MSE = 0.655; New: *F*(1,13) = 0.007, *p* = 0.936, MSE = 1.763]. ERPs for unprimed pictures were more positive than primed pictures at frontal electrode cluster [*t*(13) = 2.635, *p* = 0.021, SE = 0.72] but not at parietal electrode cluster [*t*(13) = 0.918, *p* = 0.375, SE = 0.71] for pictures with subsequent R responses. These results suggest that the priming effect during 500–700 ms was greater for pictures with subsequent R responses than pictures with subsequent K or New responses, and, hence, was predictive of priming effect on subsequent memory.

#### Test Phase

##### Basic memory effects

**FIGURE 3:**
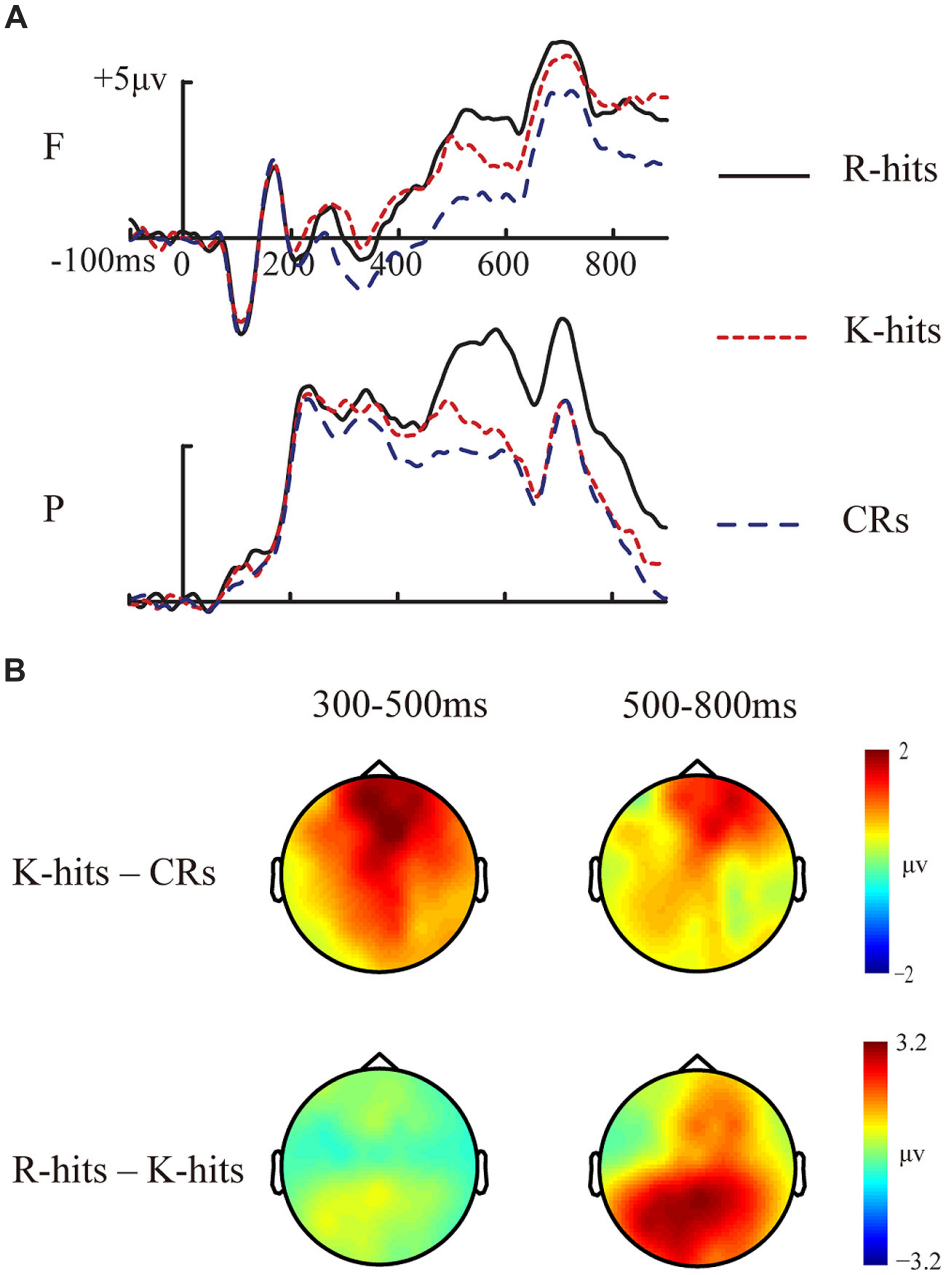
**Event-related potential waveforms and topographic maps for basic memory effects. (A)** Grand-averaged ERP waveforms for R hits, K hits, and CRs. **(B)** Topographic maps for FN400 (K hits minus CRs at 300–500 ms) and LPC (R hits minus K hits at 500–800 ms) for old/new effects. F, frontal electrode cluster; P, parietal electrode cluster.

For the analysis of primary memory effects, we collapsed ERPs across prime type and prime status to compare ERPs for R hits, K hits, and correct rejections (CRs). ERPs associated with familiarity were compared between K hits and CRs, whereas ERPs associated with recollection were compared between R and K hits. Based on previous studies (Rugg et al., 1998; Woollams et al., 2008), time windows of 300–500 ms and 500–800 ms were used to index FN400 effect and parietal LPC effect, respectively. Two-way ANOVA involving response type (R/K/CR) and electrode cluster (frontal/parietal) was conducted separately for each time interval. Grand-averaged ERP waveforms of R hits, K hits, and CRs, and topographic maps of FN400 and LPC effects are shown in **Figures 3A,B**.

###### 300–500 ms

The two-way ANOVA revealed a significant main effect of response type [*F*(2,30) = 33.652, *p* < 0.001, MSE = 0.684]. The interaction between response type and electrode cluster did not reach significant [*F*(2,30) = 3.166, *p* = 0.059, MSE = 0.528]. ERP amplitudes were not different between R and K hits (*p* > 0.1). ERP amplitudes for R and K hits were more positive than ERP amplitudes for CRs (all *p* < 0.001). The topographic map (**Figure 3B**) indicates that the FN400 effect between K hits and CRs was fronto-centrally distributed.

###### 500–800ms

The two-way ANOVA revealed a significant main effect of response type [*F*(2,30) = 16.571, *p* < 0.001, MSE = 3.692]. The interaction between response type and electrode cluster did not reach significant [*F*(2,30) = 3.058, *p* = 0.062, MSE = 1.713]. The ERP amplitudes were more positive for R hits than for K hits (*p* = 0.005) or CRs (*p* < 0.001). The ERP amplitudes for K hits were not significantly different from those for CRs (*p* > 0.1). The topographic map (**Figure 3B**) indicates that the LPC effect between R and K hits was centro-parietally distributed.

##### Effect of masked repetition priming on different type of old/new effects

**FIGURE 4:**
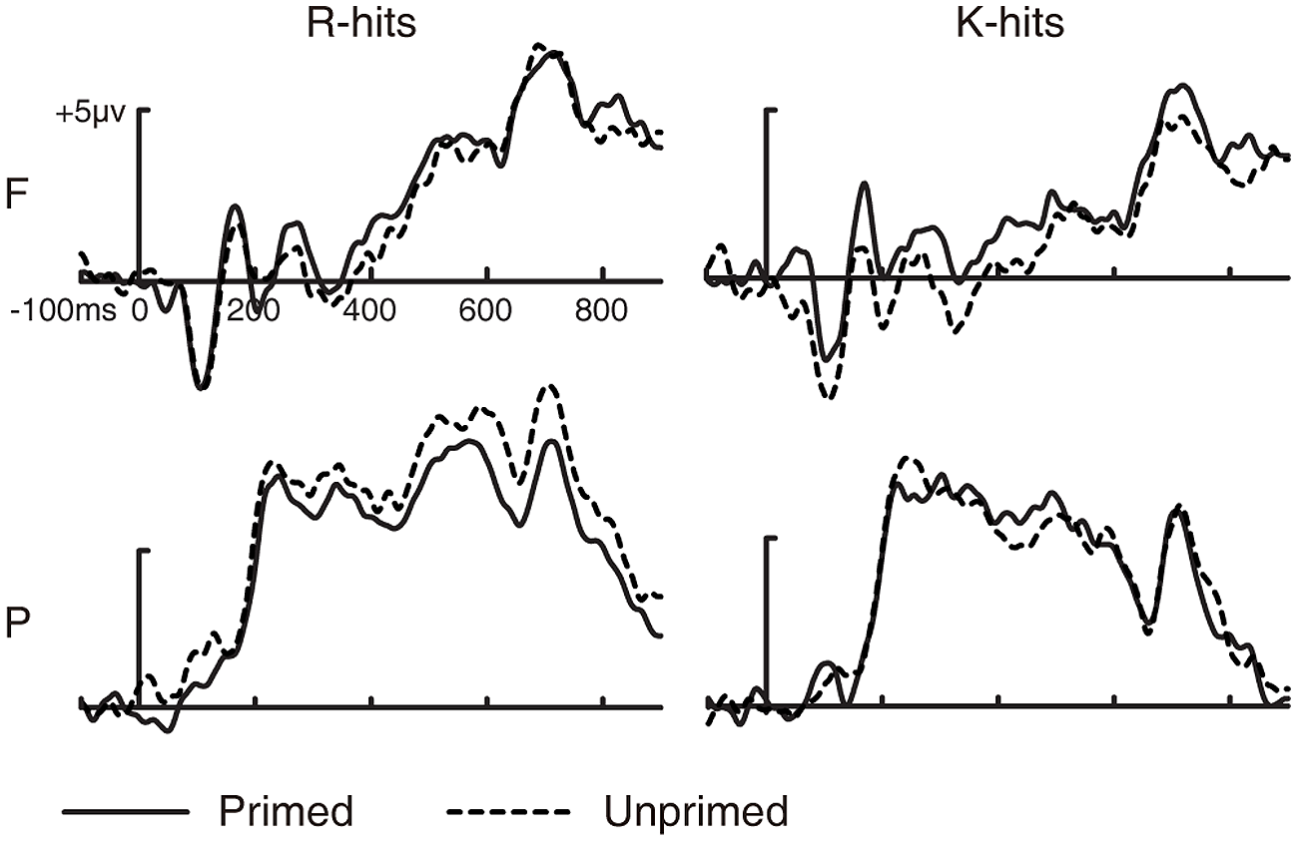
**Grand-averaged ERP waveforms for R and K hits as a function of prime status.** Grand-averaged ERP waveforms for R hits and K hits as a function of prime status. F, frontal electrode cluster; P, parietal electrode cluster.

Analysis was conducted on ERP responses to R and K hits (as a function of prime status) to examine which old/new effect was affected by masked repetition priming in the study phase. Data from one subject were excluded from this analysis because this subject had fewer than 16 artifact-free trials in one or more conditions. Three-way ANOVA involving response type (R/K), prime status (primed/unprimed in the study phase), and electrode cluster (frontal/parietal) was conducted separately for ERPs during 300–500 ms (FN400) and 500–800 ms (LPC). Grand-averaged ERP waveforms for primed and unprimed R and K hits are shown in **Figure 4**.

For FN400, the three-way ANOVA revealed a significant two-way interaction between prime status and electrode cluster [*F*(1,14) = 7.277, *p* = 0.017, MSE = 0.942]. No other two- or three-way interaction was significant (all *p* > 0.1). We collapsed the ERPs to R and K hits (i.e., old hits) to examine the interaction between prime status and electrode cluster. Amplitudes of ERPs for primed old hits were more positive than for unprimed old hits at the frontal electrode cluster [*t*(14) = 2.393, *p* = 0.031, SE = 0.372], but not at the parietal electrode cluster [*t*(14) = 0.174, *p* = 0.865, SE = 0.38]. These results indicate that masked repetition priming in the study phase increased the FN400 old/new effect.

For LPC, the three-way ANOVA revealed a significant three-way interaction [*F*(1,14) = 4.859, *p* = 0.045, MSE = 0.499]. The two-way interaction between prime status and electrode cluster was significant for R hits [*F*(1,14) = 6.772, *p* = 0.021, MSE = 1.111] but not for K hits [*F*(1,14) = 0.366, *p* = 0.555, MSE = 0.799]. Amplitudes of ERPs were more positive for unprimed than for primed R hits at the parietal electrode cluster [*t*(14) = 2.732, *p* = 0.016, SE = 0.479] but not at the frontal electrode cluster [*t*(14) = 0.213, *p* = 0.834, SE = 0.502]. These results suggest that masked repetition priming in the study phase decreased the LPC old/new effect for R hits.

## Discussion

The behavioral and ERP results of this study suggest that picture processing fluency can impair subsequent recollection, which is consistent with the findings of previous studies regarding the effect of perceptual fluency on subsequent recognition memory (e.g., Hirshman and Mulligan, 1991). Masked repetition priming in the study phase was associated with early N/P190 and late anterior LPC priming effects; however, only the LPC priming effect was predictive of the effect of processing fluency on subsequent recognition memory.

Although the impairment of processing fluency on subsequent recollection was supported by behavioral and ERP results, these two groups of results were inconsistent with regard to familiarity. The behavioral results indicated that the effect of processing fluency on subsequent familiarity was not significant, whereas the ERP results suggested that processing fluency increased subsequent familiarity (reflected by more-positive FN400 effects). The reason for this difference may be that FN400 reflects conceptual priming, rather than familiarity, as suggested recently by some researchers (Paller et al., 2007; Voss and Paller, 2007). This explanation would suggest that processing fluency actually affects subsequent conceptual priming. However, to our knowledge, no study has investigated this effect using the masked repetition priming paradigm. Thus, this interpretation should be considered with caution. Another possible reason for the difference between behavioral and ERP data is that the former were less sensitive than the latter in measuring the effect of processing fluency on subsequent familiarity. As the findings of the present study cannot be used to determine the correct explanation, further studies are needed to investigate whether processing fluency can indeed affect subsequent familiarity.

The finding that the LPC effect was larger for unprimed than for primed trials implies that LPC can be graded. Wilding (2000) found that the LPC old/new effect varied as a function of the number of correct source judgments, supporting the view that this effect reflects recollection in a graded, rather than an all-or-none, manner. These results imply that recollection is not a discrete process, supporting the viewpoint underlying the continuous dual-process signal-detection (CDPSD) model (Wixted, 2007; Wixted and Mickes, 2010), which posits that recollection and familiarity are continuous signals. In contrast, the dual-process signal-detection (DPSD) model (Yonelinas, 1994, 1997, 2001, 2002) posits that familiarity is a continuous signal-detection process, whereas recollection is a high-threshold process but not a continuous signal. However, Yonelinas et al. (2010) posited that recollection could also vary in several ways in their model, although a threshold below which recollection fails to discriminate between studied and unstudied items exists, and that this threshold nature of recollection can break down under some conditions. Thus, our finding that the LPC effect varied with recollection can also be reconciled with the DPSD model.

Masked repetition priming was associated with an earlier N/P190 effect and a later anterior LPC effect. Previous studies have suggested that N/P190 reflects early perceptual processing of object features (Eddy et al., 2006; Eddy and Holcomb, 2010). Thus, the decreased N/P190 effect for primed pictures may reflect more-fluent perceptual processing. However, the LPC effect should reflect episodic encoding activities and not processing fluency. The time window and topographic distribution were similar to those of the DM effect that reflects successful encoding (e.g., Paller et al., 1987; Nie et al., 2004), and the LPC priming effect was predictive of the effect of priming on subsequent recognition memory. Thus, the decreased LPC for primed pictures indicates that these stimuli involved less episodic encoding compared to unprimed pictures.

These interpretations suggest a possible explanation for how processing fluency impairs subsequent recollection. Increased perceptual fluency reduces the amount of perceptual features sent to medial temporal regions, where information is integrated into episodic memory (Paller and Wagner, 2002; Opitz, 2010), by facilitating early perceptual processing of object features, which might lead to reduced episodic encoding activity and result in worse recollection-based recognition memory. However, only the priming effect associated with perceptual fluency (N/P190) was observed in the present study; further studies are needed to investigate the effects of fluency at other processing levels (e.g., the semantic level, reflected by N400) on subsequent recognition memory using longer (e.g., 50 ms) masked prime picture presentation time.

Studies investigating the contribution of fluency in the test phase to recognition memory revealed that the fluency of items in the test phase affected familiarity but not recollection (e.g., Rajaram, 1993). This result is in contrast to findings from the present study, which was designed to investigate the effect of fluency on subsequent recognition memory. This discrepancy indicates that different mechanisms underlie the effects of fluency in the study phase on subsequent recognition memory and the effects of fluency in the test phase on recognition memory. The fluency attribution model has been used widely to explain the contribution of fluency to recognition memory. This model assumes that when a subject is unaware of the source of increased fluency of items in the test phase, (s)he will attribute the increase to a previous encounter with the stimuli (e.g., Jacoby and Whitehouse, 1989; Woollams et al., 2008; Lucas et al., 2012; for other alternative explanations, see Whittlesea and Williams, 1998; Huber et al., 2008). The results of our study indicated that fluency affects subsequent recollection by facilitating perceptual processing, which leads to reduced encoding activities in the study phase (reflected by the LPC priming effect).

Although the results of the present study suggest that processing fluency hinders encoding activities, processing fluency does not always lead to worse recognition memory. Some studies have shown that perceptual fluency did not affect or even improved subsequent recognition memory (Koriat, 2008; Yue et al., 2013). These inconsistent results suggest the existence of boundaries when processing fluency can affect subsequent recognition memory. The present findings suggest that processing fluency must affect encoding activities to influence subsequent recognition memory, as the priming effect associated with encoding (LPC), but not that associated with fluency (N/P190), was predictive of the effect of processing fluency on subsequent recollection. Further studies are needed to investigate factors that manipulate the relationship between fluency and episodic encoding, as well as the circumstances that determine when fluency can affect subsequent recognition memory. Studies using other paradigms (e.g., perceptual-interference) are also needed to investigate the effect of the fluency of picture stimuli on subsequent recognition memory.

Another potential caveat of our findings is that R/K responses and LPC/FN400 effects may not reflect two qualitatively distinct processes (recollection and familiarity), as suggested by single-process theories of recognition memory. Some researchers have argued that R and K responses reflect only a difference in confidence, i.e., R responses are subject to a stricter criterion than are K responses (Donaldson, 1996; Dunn, 2004, 2008). A state-trace analysis showed that FN400 and LPC effects were consistent with a single underlying variable, according to single-process models (Freeman et al., 2010). We offer the following points to alleviate these concerns. First, some recent studies have suggested that the R/K procedure can effectively distinguish familiarity and recollection when researchers ensure that participants do not confuse R and K responses with confidence ratings, by providing instructions and asking subjects to report reasons for responses of both types in the practice phase (Yonelinas and Parks, 2007; Migo et al., 2012; Wang and Yonelinas, 2012), as in our experiment. Therefore, R responses should reflect recollection in the present experiment. Second, although the specific functions of FN400 and the LPC continue to be debated, most researchers agree that they are dissociable in terms of timing and topographic distribution (Griffin et al., 2013). In addition, our results showed that the FN400 effect was increased, whereas the LPC effect was decreased, by masked repetition priming, suggesting a difference in underlying processes. However, considering current uncertainty about the relationship between R and K responses in the R/K paradigm, further studies are needed to provide clearer interpretations of the effects of fluency on recollection and familiarity.

## Conclusion

We found that processing fluency impairs subsequent recollection, even when participants are unaware of its source, and extended the effects of processing fluency on subsequent recognition memory to picture stimuli. Analysis of the ERP data suggested that perceptual fluency is associated with decreased anterior N/P190 activity. Perceptual fluency impairs subsequent recollection by reducing later episodic encoding activities, as reflected by the reduced LPC. Future studies should investigate whether processing fluency affects familiarity.

## Conflict of Interest Statement

The authors declare that the research was conducted in the absence of any commercial or financial relationships that could be construed as a potential conflict of interest.

## Footnotes

1 The same analysis conducted on the raw R and K responses revealed a significant two-way interaction [*F*(1,15) = 6.161, *p* = 0.025, MSE = 0.003]. Higher proportions of R responses, but lower proportions of K responses [*t*(15) = 2.257, *p* = 0.039, SE = 0.014], were obtained for unprimed compared to primed pictures. This finding, which is opposite to the results of the IK proportions, might result from the fact that the K responses underestimated the contribution of familiarity in the unprimed condition.

2 We also performed Bayesian analyses of the *t*-test for the effect of priming on subsequent IK responses to test whether the null effect of priming on subsequent familiarity was true (Rouder et al., 2009; Morey and Rouder, 2011) The null hypothesis (no difference between primed and unprimed IK) was hardly more likely (two times) to be true than the alternative hypothesis (Jeffreys, 1961; Kass and Raftery, 1995). Therefore, masked repetition priming might affect subsequent familiarity, but this effect was not significant in the present study.

3 N300 and N400 priming effects were reported in previous studies (e.g., Eddy et al., 2006) but were not detected in our grand-averaged ERP waveforms. Consistent with the observation of ERP waveforms, none of the main effects or interactions involving the prime status were significant. Non-significant N300 and N400 priming effects might result from the relatively shorter presentation of the masked prime pictures, as one previous study suggested that the priming effect during these time windows is more pronounced when the duration of the masked prime picture is longer (Eddy and Holcomb, 2010).
